# Tag-to-Tag Interference Suppression Technique Based on Time Division for RFID

**DOI:** 10.3390/s17010078

**Published:** 2017-01-01

**Authors:** Grishma Khadka, Suk-Seung Hwang

**Affiliations:** 1 Department of Advanced Parts and Material Engineering, Chosun University, Gwangju 501-759, Korea; grishmakhadka@chosun.kr; 2 Department of Electronic Engineering, Chosun University, Gwangju 501-759, Korea

**Keywords:** RFID, time division, interference suppression, tag-to-tag collision, signal-to-noise ratio

## Abstract

Radio-frequency identification (RFID) is a tracking technology that enables immediate automatic object identification and rapid data sharing for a wide variety of modern applications using radio waves for data transmission from a tag to a reader. RFID is already well established in technical areas, and many companies have developed corresponding standards and measurement techniques. In the construction industry, effective monitoring of materials and equipment is an important task, and RFID helps to improve monitoring and controlling capabilities, in addition to enabling automation for construction projects. However, on construction sites, there are many tagged objects and multiple RFID tags that may interfere with each other’s communications. This reduces the reliability and efficiency of the RFID system. In this paper, we propose an anti-collision algorithm for communication between multiple tags and a reader. In order to suppress interference signals from multiple neighboring tags, the proposed algorithm employs the time-division (TD) technique, where tags in the interrogation zone are assigned a specific time slot so that at every instance in time, a reader communicates with tags using the specific time slot. We present representative computer simulation examples to illustrate the performance of the proposed anti-collision technique for multiple RFID tags.

## 1. Introduction

Recent advancements in digital electronics, signal processing, and wireless communications have given rise to low-cost and low-power automatic wireless sensor systems, which allow non-contact and non-line-of-sight reading of data using electromagnetic signals. Radio-frequency identification (RFID) is an important building block for the Internet of Things (IoT), which helps to connect the status of a physical object to the Internet by capturing meaningful data in order to trace and share information [1,2]. It has become a key technology that spans a wide range of applications such as asset management, supply chains, and construction. An RFID system consists of a reader (interrogator), a tag (transponder), and middleware software that helps with data processing from the tag to the reader using RF signals [3,4]. Tags are classified as passive, semi-passive, and active depending on their functionality. Since a passive tag is not equipped with a power source, it requires energy from the reader in order to send back the re-modulated signal containing its ID information [4,5]. RFID communication based on backscatter tag signals depends on various factors including the power of the signal, the radar cross section (RCS) of the tag, and propagation loss.

For example, the RFID tags used in the construction industry for materials management suffers a lot from interference signals originating from neighboring tags, which ultimately results in the tag-to-tag collision problem. In order to minimize data collisions caused by neighboring tags, in this paper, we propose an anti-collision algorithm based on the time-division (TD) technique. In the TD technique, each tag’s response is differentiated by a specific time interval that corresponds to that particular tag for the transmission of data to the reader within the same reader-coverage area. Therefore, a data frame is divided into several timeslots, each of which is assigned to individual tags, and the reader can read all of the tags in chronological order [6]. Furthermore, the data frame considered in this paper includes the preamble frame, which helps with the channel estimation, signal-to-noise ratio (SNR) estimation, synchronization, etc. When transmitting data from a tag to the reader, the tag sends the preamble data before transmitting its main ID information to broadcast the destination of the tag information. After the reader receives the preamble data, it records TD slots for the data transmission corresponding to tags. In RFID applications, the TD method is required to group tags using the same time slot such that they only transmit within a specific time interval. In this paper, we compare the performance of the proposed TD method to the conventional method without the consideration of interference suppression and anti-collision technology and the CDMA technique based on the gold-code sequence, which spreads the tag backscatter signals to separate the signals of each individual tag. To verify the performance of our system, we perform our simulation in various reader interrogation zones.

The rest of the paper is organized as follows. Section 2 describes the RFID collision problems, and discusses and analyzes some previous works related to the collision problem. In Section 3, we present details of the received signal model employed in this study. In Section 4, we introduce the proposed TD solution for the anti-collision problem for a single reader, while in Section 5, we present a performance analysis of the proposed method in terms of bit error rate (BER) and frame error rate (FER). Finally, in Section 6, we conclude the paper.

## 2. RFID Collision Problem

In this section, we discuss types of RFID collisions and related previous works.

### 2.1. Types of RFID Collision

The occurrence of interference due to tag signals is a common issue in RFID systems. Simultaneous transmission from multiple tags leads to collision as the readers and the tags normally use the same channel. Collisions are classified as reader-to-reader, reader-to-tag, and tag-to-tag collisions [7,8].

#### 2.1.1. Reader-Reader Collision

A reader-to-reader collision occurs when neighboring readers simultaneously interrogate a specific tag in the same frequency band. Furthermore, it may occur when the tag is within the readable range of one reader and the interference range of the other reader; the signal from the latter reader may interfere with the return signal from the tag. Reader-to-reader collision is discussed in more detail in Colorwave [9,10,11,12].

#### 2.1.2. Reader-to-Tag Collision

A reader-to-tag collision (tag interference) occurs when a tag receives multiple concurrent queries from different readers. This problem can be solved by assigning different channels to nearby readers [13].

#### 2.1.3. Tag-to-Tag Collision

**Figure 1:**
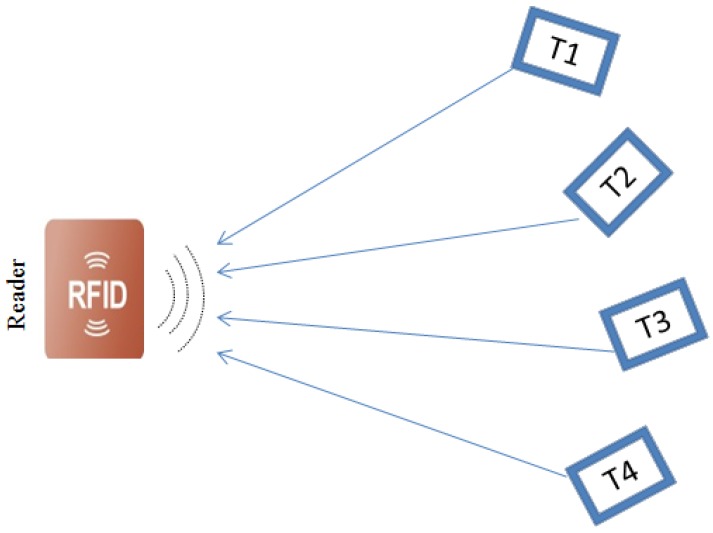
Tag-to-tag collision.

A tag-to-tag collision occurs when multiple signals simultaneously arrive at the reader, preventing the reliable detection of all tags within its interrogation zone. This scenario is illustrated in Figure 1, where tags (T1, T2, T3 and T4) simultaneously send signals to a single reader, preventing the reader from correctly recognizing a particular tag. Without an anti-collision protocol, the data from these tags would not only collide at the reader, hence degrading their identification, but will also lead to wasted bandwidth and energy [14].

### 2.2. Related Works on Collision Problem

RFID is an accurate object identification system that is used commercially to detect and manage information pertaining to various items. However, the large-scale acceptance of RFID for industrial applications is lower than expected, and this is primarily due to several issues including environmental factors, multipath fading [15], as well as the tag-collision problem. The tag-collision problem generally occurs due to a lack of proper co-ordination between the reader and tags. The backscattering response from the tag to the reader can collide with a neighboring tag, which ultimately obscures the reader from properly reading tag IDs.

In this paper, we focus on the problem of a single reader in an environment with multiple tags. Further, we focus on the practical application of RFID technology to the construction industry. Typically, on a construction site, there are a large number of tagged objects. Thus, it is extremely important to simultaneously improve the object identification performance and minimize operating costs. A variety of multiple access technologies have been used in recently developed RFID systems such as space-division multiple access (SDMA), frequency-domain multiple access (FDMA), time-division multiple access (TDMA), and code-division multiple access (CDMA), which is known as spread-spectrum [16,17,18].

SDMA reuses certain resources (channel capacity) in spatial slots. SDMA protocols separate the channel using electronically controlled directional antennas or multiple readers to identify tags. However, drawbacks of this approach include the high implementation cost, the need to design a complicated antenna system, and its limitation to only a few specialized applications. On the other hand, FDMA uses different frequency ranges for data transmission to and from the transponders, which result in a high cost to implement the reader. This requires a complex receiver at the reader end because a dedicated receiver must be provided for each reception channel. Although CDMA exhibits good performance for multiple tags, it requires that their data be multiplied by a pseudo-random noise (PN) sequence before transmission. On the contrary, the TDMA anti-collision algorithm allocates several timeslots in each frame to solve the collision problem. The TDMA anti-collision technique is the most popular RFID reader-to-reader anti-collision scheme, and it is used in the Colorwave algorithm [19,20], where each reader chooses a random time slot to transmit data. The TDMA anti-collision method chronologically divides the total available channel capacity among participants. Its procedures are classified as synchronous reader-driven and asynchronous tag-driven. In the reader-driven procedure, at a particular time interval, an individual tag is first selected from a group of tags using specific algorithms, and then a data communication link is made between the selected tag and reader. On the other hand, with the tag-driven procedure, the tag announces itself to the reader by transmitting its IDs in the presence of a reader. The former and latter procedures are also called reader-talk-first (RTF) and tag-talk-first (TTF), respectively. TTF is relatively slow and inflexible compared to RTF [16,21,22].

For anti-collision algorithms in the RFID system, Abramson’s Logic of Hiring Access (ALOHA) protocol and binary tree algorithms, which are based on deterministic and probabilistic approaches, are widely used. Most of the standards employed for ultra-high frequency (UHF) RFID systems refer to binary tree searches for anti-collision algorithms as deterministic schemes because each roof-to-leaf path denotes a unique tag ID, and it is possible to simultaneously retrieve all IDs after all branches are completely searched. On the other hand, the ALOHA anti-collision algorithm is usually referred to as a probabilistic scheme, where each tag ID will have a probability of successful retrieval [23].

Tree-based anti-collision algorithms use a virtual tree to read all tag data within the read range of the reader by iteratively querying a subset of tags at different levels, and tag IDs are distributed based on their prefixes. The root is the set of tags to be identified, intermediate nodes represent the group of colliding tags, and the leaves represent individual tag responses [24]. They are further classified as tree splitting (TS), query tree (QT), binary search (BS), and bitwise arbitration (BTA) [17]. In the TS protocol, multiple responding tags are grouped using a random number generator. On the other hand, the QT protocol stores the tree information at the reader. The reader broadcasts a query with a bit-string prefix, which is then matched with the tag ID’s prefix, after which the tag with the matched prefix responds to the query with its ID to the reader. This process continues until only one tag responds [25]. This overcomes the problem of generating a random number using TS, and decreases the cost and computational complexity. In BS, the communication starts by the reader transmitting the serial number of tags. The serial number is then compared against their ID. In this protocol, communication is possible if tags with an ID equal to or lower than the serial number respond to the reader. Unlike the above protocols, BTA operates by requesting tags to respond bit-by-bit from the most significant bits to the least significant bit of their ID [17].

The ALOHA protocol is a probabilistic approach, where a signal responds within a random time slot in a frame to identify tags. Of the different ALOHA-based protocols, the frame-slotted ALOHA (FSA) and dynamic frame-slotted ALOHA (DFSA) are most commonly used in RFID systems because they can reduce the collision probability between tags. In FSA, the reader determines the frame size and the actual duration of the slot, and tags use random slots from among the entire set of slots (the total number of slots and the frame size are fixed). Therefore, in a reader interrogation zone, for a small number of tags, there is the problem of wastage because of the empty slots, and for a large number of tags, there is a collision problem. This drawback of FSA is partially solved by the DFSA. In DFSA, the reader will add the number of slots in one frame when there a large number of slots have collided. Similarly, the reader will decrease the number of slots in one frame size when there are many empty slots [26]. In DFSA, the size of the next frame is adjusted after estimating the number of remaining tags at the end of each frame without performing any operation for the ideal slots and collided time slots within the current frame. Therefore, the request time by the reader during these time slots are wasted. Consequently, if we try to reduce the request time, the probability of tag collisions may be increased [27].

## 3. Received Signal Model

The received signal, r(k), at a sample index k can be modeled as

(1) $$r\left(k\right)=x_{1}\left(k\right)+x_{2}\left(k\right)+x_{3}\left(k\right)+\ldots +x_{L}\left(k\right)+n\left(k\right),$$

which can be summarized as

(2) $$r\left(k\right)=\overset{L}{\underset{l=1}{\sum }}x_{l}\left(k\right)+n\left(k\right)\;$$

where l represents the *l*th tag, L is the total number of tags transmitting the signal to the reader, $x_{l}\left(k\right)$ is the transmitted signal of the *l*th tag, and n(k) is the additive white Gaussian noise (AWGN) with independent and identically distributed components with zero mean and variance $\sigma^{2}$.

Using Friis’ free-space equation, the relationship between the signal strength and distance, and between the received power at the antenna of the tag, $P_{tag},$ and the transmitted power from the reader, $P_{r}$ is given by

(3) $$P_{tag}=P_{r}G_{r}G_{tag}{\left(\frac{\lambda }{4\pi d}\right)}^{2}$$

where $G_{r}$ and $G_{tag}$ are the transmitter and receiver antenna gains, respectively, λ is the wavelength (m), and d is the distance between the transmitter and the receiver antennas [28,29]. Equation (3) can be expressed in decibel form as

(4) $$P_{tag}\left[dB\right]=P_{r}\left[dB\right]+G_{r}\left[dB\right]+G_{tag}\left[dB\right]\;-10log_{10}{\left(\frac{4\pi d}{\lambda }\right)}^{2}$$

which describes the amount of power available to the RFID tag for operation. Note that in this paper, [dB] refers to the decibel scale of the specific value. Since the power of the radio-frequency signal decreases when it propagates into space, there is a difference between the power delivered from the transmitting antenna and the power obtained at the receiving antenna, and this is known as the path loss [30,31]. In Equation (4), $10log_{10}{\left(\frac{4\pi d}{\lambda }\right)}^{2}$ is the free-space path loss factor, and is defined as $\;P_{loss}\left[dB\right]$.

The decreasing trend of the signal in terms of the log-distance path loss is mathematically modeled as [32]

(5) $$P_{loss}\left(dB\right)=P_{loss}\left(r_{0}\right)+\;10nlog\left(\frac{d}{r_{0}}\right)$$

where $r_{0}$ is a reference distance, $P_{loss}\left(r_{0}\right)$ is the free-space path loss at a distance $r_{0},$ n is a value that depends on the surroundings and building type (a more hostile environment results in a higher value of n), and the path loss; accordingly, it is higher for the same distance when compared to an environment with a lower value of n [33,34,35].

## 4. Anti-Collision for RFID Based on TD

In this section, we propose an efficient and simple anti-collision RFID system that is specifically for smart green construction technology. The goal of this construction technique is to reduce the construction cost and to enhance the speed of construction by reusing and recycling the materials used in a previous building. In order to improve the traditional construction technology, the smart green construction generally employs a construction management system that is based on a sensing technique such as RFID. In this case, RFID provides the vital roles for managing a variety of construction materials, monitoring the status of new or reused materials, and classifying the generation of each construction materials. On the construction site, the tracking and storing of materials’ information using RFID remain challenging because a large number of materials are used in narrow spaces. In this case, we need to employ a low-cost RFID system utilizing a simple protocol, unlike the ALOHA system or gold-code based system, and it should have a good anti-collision performance. In addition, the RFID tag does not need to adaptively allocate time slots in a data frame, as is the case with ALOHA, because construction materials may not move for a relatively long time.

In this paper, we propose a simple and efficient RFID system that is based on the TD method. The proposed RFID tag transmits data using the specific time slot, and this time slot is fixed. Before tags are attached to construction materials, the tags are grouped according to their time slots (note that the number of groups may be determined according to the size of the building or the amount of construction materials). When we attach tags with specific time slots to the construction materials, they should be managed to avoid that tags that are in the same group are located in a neighbor. For example, if the tag of the first group is attached to a specific construction material, tags belonging to other groups should be attached to the neighboring materials. This process of managing the locations of RFID tags is simple because construction materials are generally on the same site for relatively long periods of time until the building is demolished and the materials reused in another building. Although we focus on an application of the proposed RFID system on the construction site in this paper, it might be applied to other areas, where objects are placed in the same site for relatively long periods, such as the storage, library, and industrial sites with various devices installed.

The proposed anti-collision RFID algorithm focuses on the case involving a single handheld reader and multiple tags. In addition to efficiently reducing the probability of collisions due to other tags by assigning tags to previously allocated time slots to transmit data, it also employs a simple RFID protocol for one-way communication. This simple RFID protocol may reduce the implement cost, because the RFID tag transmits data using the fixed specific time slot, without the complicated mathematical operation process, unlike the anti-collision RFID system based on the gold-code or ALOHA. Note that the gold-code anti-collision RFID tag requires a significant amount of numerical operations including the multiplication and addition, and the ALOHA technique requires an adaptive resource process for allocating time slots within a data frame.

### 4.1. RFID Signal Power Model Considering Interference Signals

In RFID systems, once the tag is powered up by the transmitted power from the reader, the selected tag starts to respond to the reader. Since the modulated signal scattered from the RFID tag suffers from noise and interference signals that originate from other tags, the received signal at the RFID reader, which is given by Equations (1) and (2), consists of the modulated backscattered signal of interest, noise, and interference signals [36,37]. At the reader, the amount of power available to the receiver antenna for it to operate is given by

(6) $$\begin{array}{ll} P_{R-receiver}\left[dB\right] & =P_{r}\left[dB\right]+G_{r}\left[dB\right]+G_{R-\mathrm{receiver}}\left[dB\right]+10log_{10}\left(\frac{\sigma }{4\pi }\right)\\ & \;-20log_{10}\left(\frac{4\pi }{\lambda }\right)-\;40log_{10}\left(d\right), \end{array}$$

where $P_{R-receiver}\left[dB\right]$ is the power received by the receiver antenna of the reader, $G_{R-receiver}$ is the gain of the receiver antenna at the reader (which is equal to the transmitter antenna gain at the reader), and σ is the RCS of the RF tag, which is important for the system-level efficiency and reliability [35,36]. The RFID tag’s cross-section area can be written as [38,39]

(7) $$\sigma_{antenna}=\frac{{\left|\tau \right|}^{2}{G_{tag}}^{2}\lambda^{2}}{4\pi }$$

where τ is the impedance-matching coefficient between the tag chip and its antenna. Substituting the value of $\sigma_{antenna}$ into Equation (6), the modulated power from the RF tag to the reader becomes:

(8) $$\begin{array}{cc} P_{R-receiver}\left[dB\right] & =P_{r}\left[dB\right]+2G_{r}\left[dB\right]+2G_{tag}\left[dB\right]+20log_{10}\left(\tau \right)\\ & -\;40log_{10}\left(\frac{4\pi }{\lambda }\right)-40log_{10}\left(d\right). \end{array}$$

Equation (8) can be expressed in a general form as

(9) $$P_{R-receiver}=\frac{P_{r}G_{r}^{2}G_{tag}^{2}\tau^{2}\lambda^{4}}{{\left(4d\pi \right)}^{4}}$$

Equations (8) and (9) show that the total energy consumption of passive RFID systems depends on the power transmitted from the reader to the tag. Since those equations show that the effective distance r between the tag and the reader can be adjusted by maintaining the power supplied at the reader antenna, the total backscattering power of the passive RFID tag and interrogation range depends on the transmission power level of the reader. In our computer simulations, we used the above-mentioned RFID signal power models.

### 4.2. RFID Anti-Collision Scheme Based on TD

In order to suppress the interference signals from surrounding multiple RFID tags, we propose an anti-collision method based on the TD technique that allocates the tag information on the specific time slot. During reader-to-tag communication, the reader transmits a carrier signal with transmit power $\;P_{r}$ to a set of arbitrary multiple tags within their interrogation zone. If the power received by the reader is greater than the minimum power received from the tag, which is known as the reader sensitivity, data transmission may be possible from the tag to the reader. All of the tags in the coverage area of the reader then become activated and transmit their identification information to the reader with a power of $\;P_{R-receiver}$ whenever the energy received by the reader is higher than an activation threshold. If the measured $\;P_{R-receiver}$ value is higher than the activation threshold, the transmitting signal is effective for transmitting its payload. On the contrary, if the measured value is lower than the activation threshold, the transmitting signal does not effectively transmit its payload and the data transmission may fail. In the proposed RFID system, while the particular tag using the specific time slot transmits data to the reader, other tags using other time slots do not transmit any data, but maintain their sequence order. Since tags transmit data according to their turn, in the reader, interference signals from other tags are efficiently suppressed and the communication reliability is enhanced compared to conventional RFID system which has the low performance of reading capability for multiple tags, because they do not have the interference suppression or anti-collision technique.

**Figure 2:**
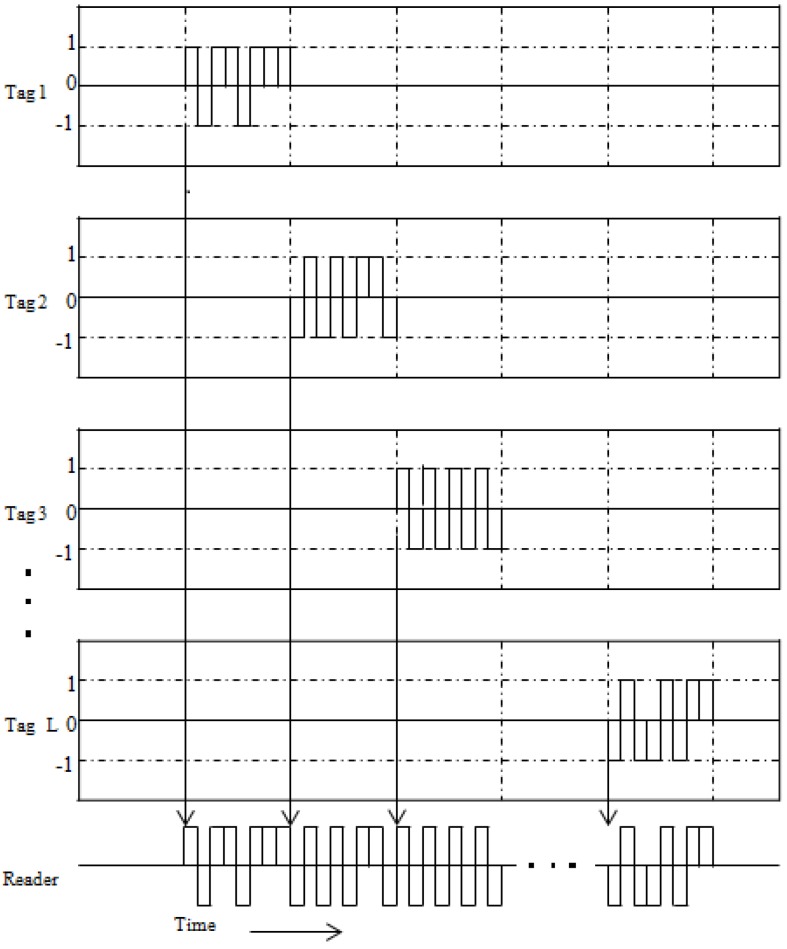
Example of transmitted signals at tags and received signal at the reader in the proposed BPSK-based RFID system.

Figure 2 shows an example of the transmitted signals at the tags and the received signal at the reader based on binary phase shift keying (BPSK), which uses a constant carrier signal amplitude with an independent data modulation technique and helps to provide stable power transfer in wireless communication [40,41,42]. In the figure, to explain the proposed system, we assume that each tag employs a unique time slot. Although in a real system, some tags can be allocated into the same time slots, interference signals from other tags are effectively suppressed compared to conventional RFID systems. We discuss the interference suppression performance for the proposed RFID system based on TD by performing computer simulations.

### 4.3. Data Architecture for RFID Anti-Collision Based on TD

In this subsection, we propose an efficient data architecture that is based on TD to prevent RFID collisions. Here, we allocate specific time slots in the RFID data frame, and a fixed number of data bits for the preamble data and the payload. Within a particular time interval, the reader recognizes data bits from a particular tag, which consists of preamble data followed by the payload. In order to prevent collisions between tags in the same reader interrogation zone, a specific time interval is reserved for the specific tags.

The functions of the preamble and payload are summarized as follows:

#### 4.3.1. Preamble

In a noisy and interference environment that may prevent us from accurately detecting the desired signal, the preamble helps to prevent performance degradation of the signal detection [43]. Thus, preamble data are used for the channel estimation, SNR estimation, synchronization, etc. At the receiver of the reader, the efficient channel and SNR estimations should be obtained to enable the accurate detection of the transmitted data from the RFID tags and the estimation of the number of tags in the interrogation zone, respectively. In addition, perfect synchronization is required for the proposed RFID approach because the tag transmits data using a specific time slot. In this paper, we assume that the channel estimation, SNR estimation, and synchronization are performed at the receiver of the reader.

**Figure 3:**
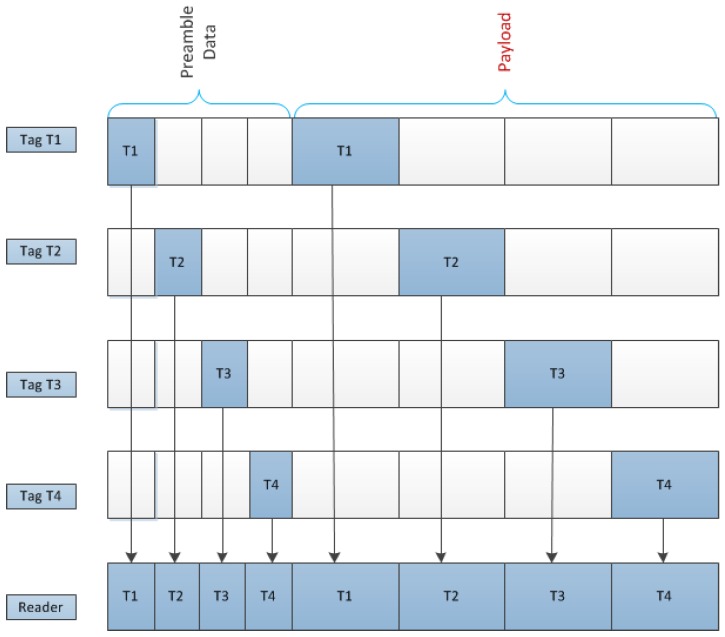
Data architecture for proposed anti-collision RFID system.

Assuming that there are four tags that each employs unique time slots, Figure 3 shows the data architecture for the proposed anti-collision RFID system. In this system, preamble data in the *l*th tag are transmitted using the *l*th time slots in the figure before transmitting the payload.

#### 4.3.2. Payload

In this RFID system, when the reader sends the wake-up signal to the tags (for simplicity, we did not include the wake-up signal in the data architecture), tags sequentially transmit the preamble and payload using the specific time slot shown in Figure 3. The payload of this RFID system includes tag information such as the identification (ID) number, electronic product code (EPC) number, and other important information [44,45].

Using the proposed data architecture, when a specific tag transmits the data to a reader, no other tags transmit any data, as shown in Figure 3. Therefore, there is no interference signal when simultaneously communicating between multiple tags and a reader. While the TD-based anti-collision architecture for RFID in Figure 3 considers four tags and a reader, it may be extended for more tags. As mentioned previously, in the case where some tags can be allocated into the same time slots in a real system, interference from other tags are effectively suppressed, compared to the case with a conventional RFID system without the anti-collision technique.

## 5. Computer Simulations

In this section, we present the computer simulation results obtained to illustrate the performance of the proposed RFID system compared to that of the conventional system without the anti-collision technique and anti-collision RFID systems that are based on the gold-code sequence [46,47]. For the simulation, we considered three data frame sizes, 96 bits, which is the minimum length required for the International Standards Organization (ISO) standard [48], 144 bits, which is the recommended frame length in the ISO standard, and 512 bits, which is the maximum frame length used in typical passive RFID systems in the ISO standard. In addition, we assume that the length of the preamble is 16 bits, which is the length generally used in RFID systems [28,48]. For the simulation involving the gold-code RFID system, we assume that the length of the gold code is seven.

These simulations focus on determining the suppression performance for interference signals from circumjacent tags within the corresponding reader interrogation zone. As mentioned in the previous section, for this simulation, we assume that the channel estimation, SNR estimation, and synchronization are perfectly performed at the receiver of the reader.

### 5.1. Four Tags with Unique Time Slots

In this subsection, to illustrate the interference suppression performance of the proposed system, we present simulation results based on four tags with unique time slots in a reader interrogation zone. For this simulation, we considered four RFID tags located at different distances within the reader interrogation zone. Moreover, we employed passive RFID tags (T1, T2, T3 and T4) with a frequency of 900 MHz and a fixed transmitted power for a distance of 2 m with a minimum loss in each bit. After we present the results for only the four tags considered without any other tags, we present other results for the same four tags as well as many other circumjacent tags within the reader interrogation zone.

**Figure 4:**
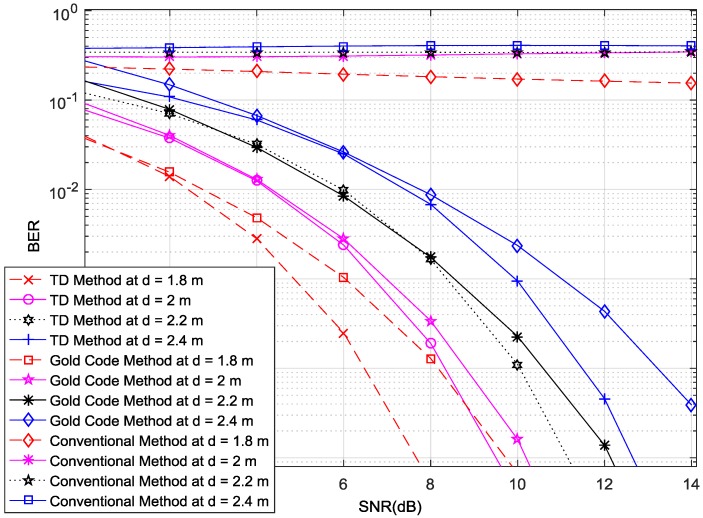
Bit Error Rate (BER) curves of the proposed system, conventional system without anti-collision technique, and anti-collision system based on the gold-code sequence.

Figure 4 shows the variation of the BER with different SNR values. There are twelve curves because the four tags are at different distances (1.8–2.4 m), for three different systems, namely the proposed system, a conventional system, and an anti-collision RFID system that is based on the gold-code sequence. In Figure 4, we observe that the BER curves for the proposed system are much lower than those of the conventional system, and are slightly lower than those of the gold-code system, which means that the performance of the proposed system is better than that of both the conventional and gold-code systems. We also observe that the conventional system does not work properly when there exist interference signals from other tags, and when the distance between a tag and the reader is not close enough. Although the gold-code system has an anti-collision performance that is similar to that of the proposed system, it requires a complicated protocol, which results in an increased cost. If we increase the length of the gold code, the anti-collision performance is improved, but the computational complexity is higher, which results in a long tag-response time.

**Figure 5:**
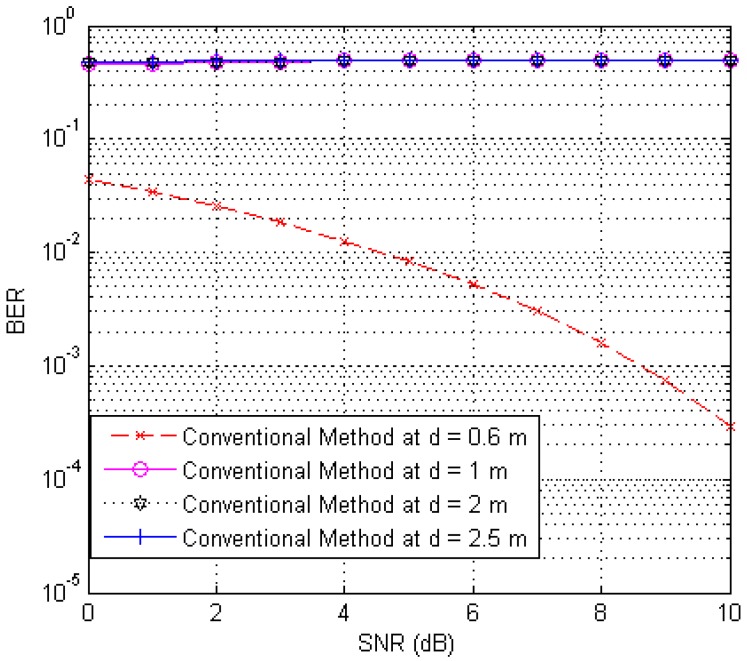
BER curves of the conventional system for four tags with different distances.

Figure 5 illustrates the case where the conventional system works properly in an interference environment. The distances between tags and the reader are 0.6, 1, 2 and 2.5 m, respectively. Moreover, we observe that only the tag at a distance of 0.6 m works properly, but other tags do not work because their BER is too high for all SNRs; this is due to the interference signals from other tags. On the other hand, the proposed system works properly for all tags in a similar case.

**Figure 6:**
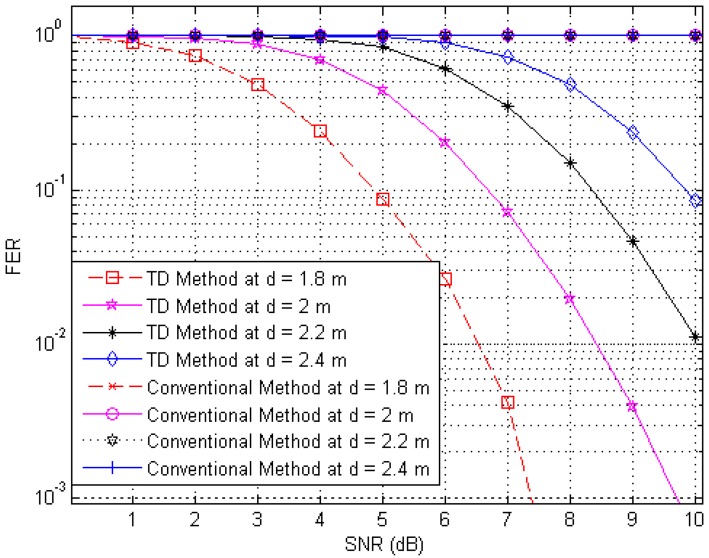
FER curves of the proposed and conventional systems with a frame length of 96 bits (distances of 1.8, 2, 2.2 and 2.4 m).

**Figure 7:**
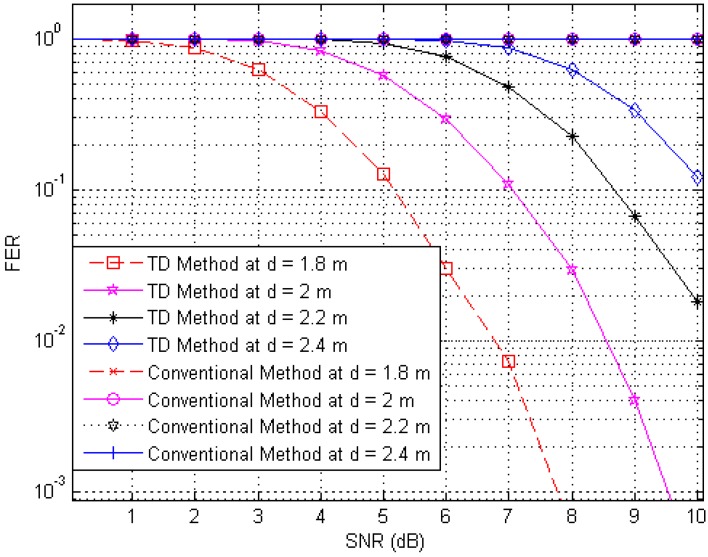
FER curves of the proposed and conventional systems with a frame length of 144 bits (distances of 1.8, 2, 2.2 and 2.4 m).

**Figure 8:**
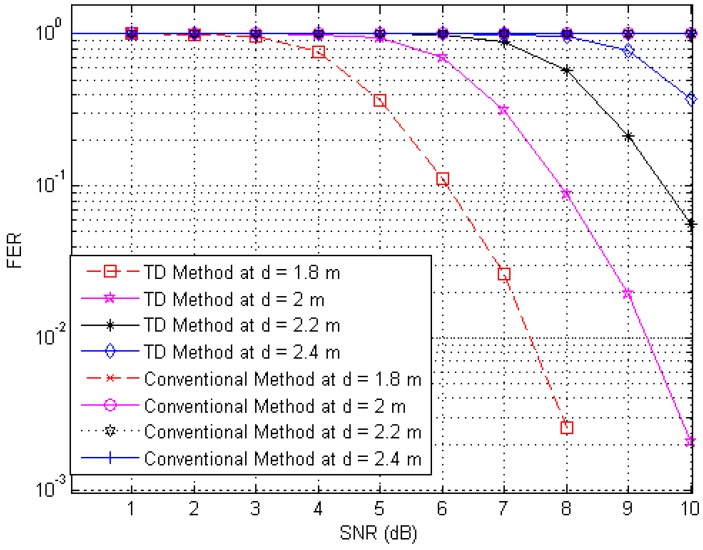
FER curves of the proposed and conventional systems with a frame length of 512 bits (distances of 1.8, 2, 2.2 and 2.4 m).

**Figure 9:**
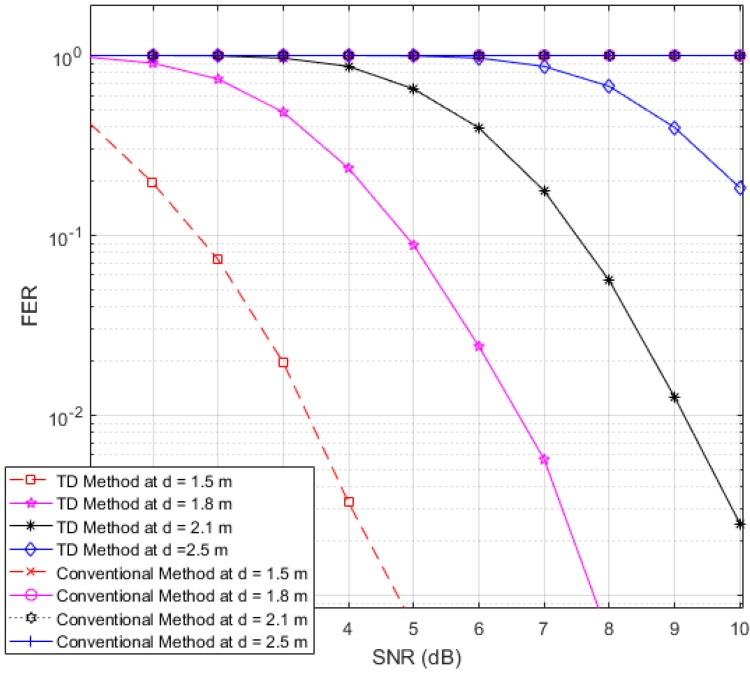
FER curves of the proposed and conventional systems with a frame length of 96 bits (distances of 1.5, 1.8, 2.1 and 2.5 m).

**Figure 10:**
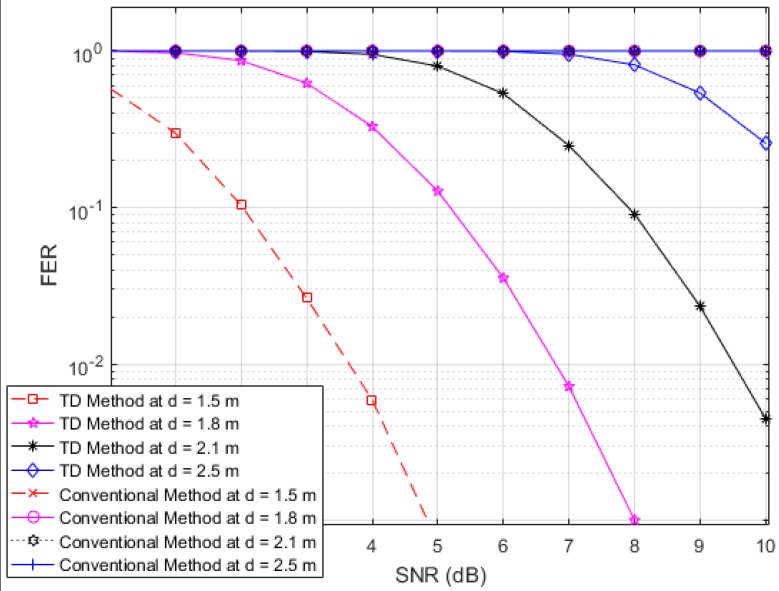
FER curves of the proposed and conventional systems with a frame length of 144 bits (distances of 1.5, 1.8, 2.1 and 2.5 m).

**Figure 11:**
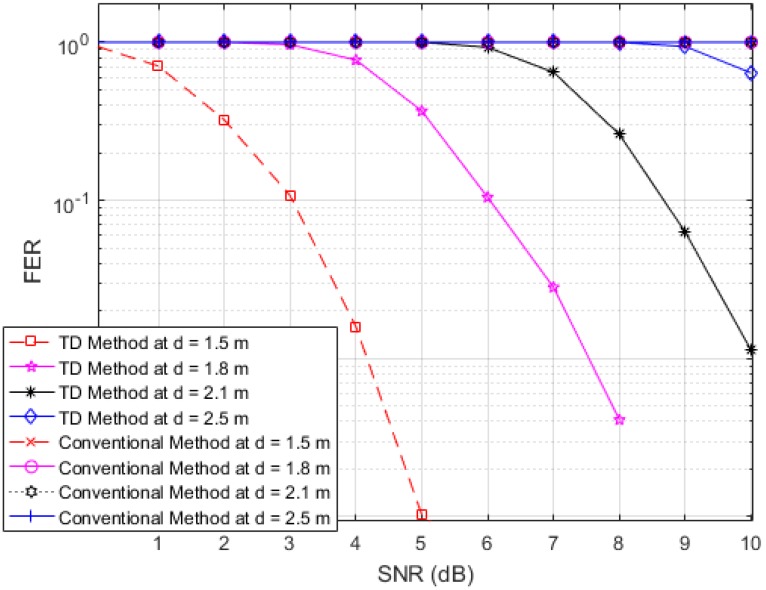
FER curves of the proposed and conventional systems with a frame length of 512 bits (distances of 1.5, 1.8, 2.1 and 2.5 m).

Next, to illustrate the interference suppression performance of the proposed RFID system compared to the conventional system, we present the simulated results for the variation in the FER as the SNR is varied in cases involving various frame lengths. Figure 6, Figure 7 and Figure 8 show FER curves for data frame lengths of 96, 114 and 512 bits, respectively, employing four tags with distances of 1.8, 2, 2.2 and 2.4 m. In addition, Figure 9, Figure 10 and Figure 11 show FER curves for the same frame lengths as in previous figures, but they employ four tags with distances of 1.5, 1.8, 2.1, and 2.5 m. In these figures, it appears that there is a single FER curve for the conventional system because the FER values for all tags are close to 0.5, which means that the reader cannot distinguish the tag identification information from the received data due to the presence of interference signals. On the other hand, in all cases, the reader that employs the proposed system correctly distinguishes the identification information for each tag. Note that in all figures, the FER for the proposed RFID system increases as the length of the data frame is increased.

### 5.2. Many Tags with Random Time Slots

Finally, in order to illustrate the interference-suppression performance of the proposed RFID system compared to the conventional system and an anti-collision RFID system based on the gold-code sequence, we present simulation results based on the use of a large number of tags with random time slots in a reader interrogation zone.

**Figure 12:**
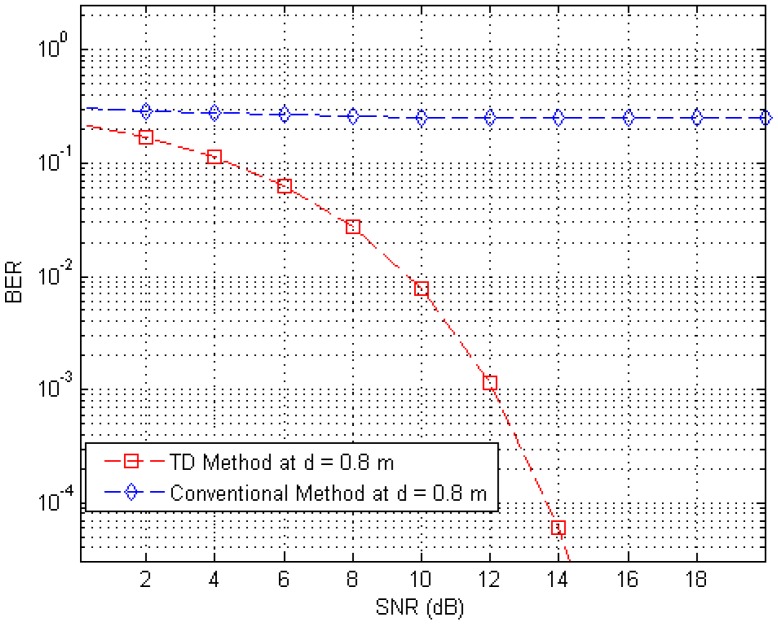
BER curves of the proposed and conventional systems at a distance of 0.8 m in the presence of 250 interference tags.

Figure 12 shows BER curves (at a distance of 0.8 m) for different SNR values with the proposed RFID system, the conventional system without the anti-collision technique in the presence of 250 interference tags (30 tags at distances ranging from 1.5 m to 4 m, 80 tags at distances ranging from 4 m to 7 m, and 140 tags at distances ranging from 7 m to 10 m) in an interrogation zone. In Figure 12, we observe that the proposed RFID system has a much better performance than the conventional system, because the BER curve of the proposed system is much lower than that of the conventional system.

**Figure 13:**
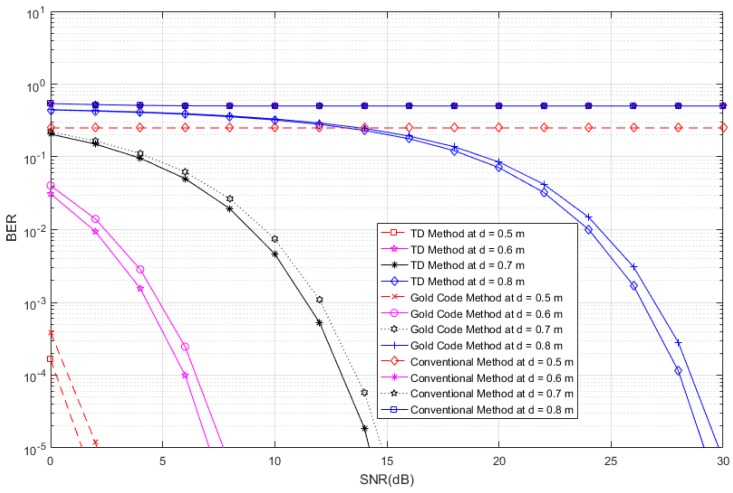
BER curves of the proposed system, conventional system without anti-collision technique, and anti-collision system based on the gold-code sequence, for four tags of interest with different distances in the presence of 510 interference tags.

**Figure 14:**
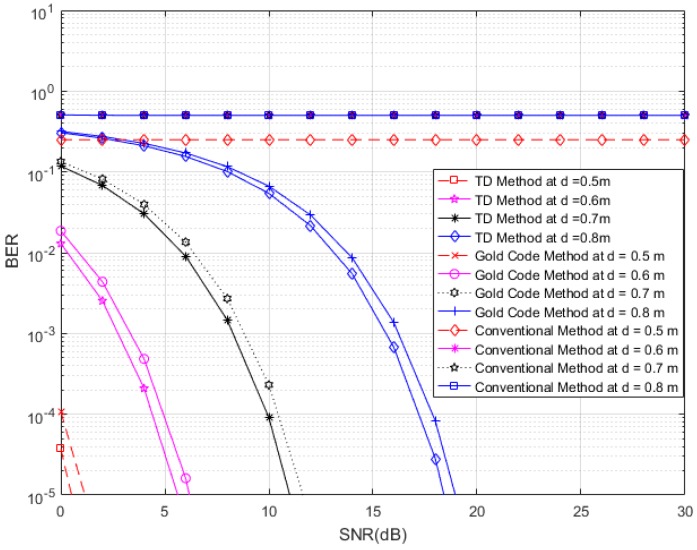
BER curves of the proposed system, conventional system without anti-collision technique, and anti-collision system based on gold-code systems, for four tags of interest with different distances in the presence of 270 interference tags.

In Figure 13, we consider four tags of interest, which have different distances (0.5, 0.6, 0.7 and 0.8 m) and unique time slots in the presence of 510 interference tags (60 tags at distances ranging from 1.5 m to 4 m, 150 tags at distances ranging from 4 m to 7 m, and 300 tags at distances ranging from 7 m to 10 m) in an interrogation zone. Similarly, in Figure 14, we consider four tags of interest, which have different distances (0.5, 0.7 and 0.8 m) and unique time slots, in the presence of 270 interference tags (30 tags at distances ranging from 1.5 m to 4 m, 90 tags at distances ranging from 4 m to 7 m, and 150 tags at distances ranging from 7 m to 10 m) in an interrogation zone. In Figure 13 and Figure 14, we compare the performance of the proposed RFID system to the conventional system and an anti-collision RFID system based on the gold-code sequence. From both figures, we observe that the proposed RFID system works properly in the interference environment for all cases and it has better performance than that of the gold-code anti-collision RFID system, while the conventional system without the anti-collision technique has high BERs for all SNRs in all cases. The SNR analyses for results of Figure 13 and Figure 14 are shown in Table 1 and Table 2, respectively. For these analyses, we consider the data reliability rates at 90%, 95%, 97%, and 99% at different distances and we indicate the minimum SNRs for achieving the considered data reliability rates in Table 1 and Table 2 for Figure 13 and Figure 14, respectively, for the proposed RFID systems, the gold-code anti-collision RFID system, and the conventional system. From both Tables, we observe that the required minimum SNRs for achieving all reliability rates for the proposed RFID system are lower than them of the gold-code anti-collision RFID system in both cases, while the conventional system does not make the considered data reliability rates for all SNRs in both cases.

## 6. Conclusions

In order to minimize the number of tag-to-tag collisions for RFID systems, we propose an anti-collision technique and data structure that employ the TD technique. In the proposed system, since only specific tags transmit the data to the reader in a specific time slot, interference signals from other tags are effectively suppressed. Therefore, when we simultaneously employ multiple tags and a single reader on an industrial site such as a construction site, the proposed RFID architecture improves the data reliability and performance, enabling the identification of information for specific objects or materials. We illustrated the interference suppression performance of the proposed RFID system by performing computer simulations, and compared the results with a conventional system without the anti-collision technique and an anti-collision RFID system that is based on the gold-code sequence. In the future, we will explore in greater detail some practical applications of the proposed RFID system.

## Figures and Tables

**Table 1:** SNR analysis at different distances in the presence of 510 interference tags.

Data Reliability Rate	Minimum SNR (dB) for TD Method at Distance (m)				Minimum SNR (dB) for Gold Code Method at Distance (m)				Minimum SNR (dB) for Conventional Method at Distance (m)			
	0.5 m	0.6 m	0.7 m	0.8 m	0.5 m	0.6 m	0.7 m	0.8 m	0.5 m	0.6 m	0.7 m	0.8 m
90%	<0	<0	4.0	18.8	<0	<0	4.4	19.3	N/A	N/A	N/A	N/A
95%	<0	<0	6.0	21.0	<0	<0	6.5	21.7	N/A	N/A	N/A	N/A
97%	<0	0.1	7.1	22.3	<0	0.7	7.9	22.8	N/A	N/A	N/A	N/A
99%	<0	2.0	9.0	24.0	<0	2.4	9.6	24.5	N/A	N/A	N/A	N/A

**Table 2:** SNR analysis at different distances in the presence of 270 interference tags.

Data Reliability Rate	Minimum SNR (dB) for TD Method at Distances (m)				Minimum SNR (dB) for Gold Code Method at Distances (m)				Minimum SNR (dB) for Conventional Method at Distances (m)			
	0.5 m	0.6 m	0.7 m	0.8 m	0.5 m	0.6 m	0.7 m	0.8 m	0.5 m	0.6 m	0.7 m	0.8 m
90%	<0	<0	0.6	8.0	<0	<0	1.1	8.6	N/A	N/A	N/A	N/A
95%	<0	<0	2.8	10.2	<0	<0	3.4	10.9	N/A	N/A	N/A	N/A
97%	<0	<0	3.9	11.2	<0	<0	4.5	12	N/A	N/A	N/A	N/A
99%	<0	0.4	5.9	13.1	<0	0.9	6.4	13.9	N/A	N/A	N/A	N/A
